# Developing Tobacco Risk Communications for Young Adults Susceptible to Dual Use of Combustible Cigarettes and Nicotine Vapes: Mixed Methods Study

**DOI:** 10.2196/89822

**Published:** 2026-08-18

**Authors:** Emma E Jankowski, Alysha C Ennis, Grace Turk, Hayley Curran, Darren Mays, Phoenix A Matthews, Michael D Slater, Theodore Wagener, Amy K Ferketich, Joanne G Patterson

**Affiliations:** 1 Department of Epidemiology, College of Public Health, The Ohio State University, 1841 Neil Ave, Columbus, OH, 43210, United States, 1 (614) 292-8350; 2 Department of Health Behavior and Health Promotion, College of Public Health, The Ohio State University, Columbus, OH, United States; 3 Center for Tobacco Research, Comprehensive Cancer Center, The Ohio State University, Columbus, OH, United States; 4 Division of Medical Oncology, Department of Internal Medicine, College of Medicine, The Ohio State University, Columbus, OH, United States; 5 School of Nursing, Columbia University, New York, NY, United States; 6 School of Communication, College of Arts and Sciences, The Ohio State University, Columbus, OH, United States

**Keywords:** young adult, LGBTQ+, lesbian, gay, bisexual, transgender, and queer, tobacco, nicotine, communication, dual use

## Abstract

**Background:**

Dual use of combustible cigarettes and nicotine vapes is disproportionately high among lesbian, gay, bisexual, transgender, and queer (LGBTQ+) young adults. Mass-reach health communications may be effective at curbing dual use. Current research is exploring whether comparative risk messaging, which presents nicotine vapes as less harmful than cigarettes, reduces dual use.

**Objective:**

This formative message testing study focused on communicating the health risks of cigarette smoking and nicotine vaping use to young adults susceptible to dual use of these products, including LGBTQ+ young adults.

**Methods:**

Online focus groups were conducted with young adults to develop candidate messages (N=12). Interviews (N=13) qualitatively explored thematic content. An online rating survey (N=286) quantitatively assessed perceived message effectiveness (PME) of and reactance to candidate messages applying standard and comparative risk message framing, compared to adapted regulatory warnings used by the US Food and Drug Administration.

**Results:**

Qualitatively, interview and focus group participants found messages featuring novel information, including toxic constituents and physical harms (eg, hypertension), most effective. “Known” harms (eg, cancer) were described as effective by LGBTQ+ young adults due to the “shock value” of fear appeals. However, participants recommended pairing “known” harms with novel information; for example, addiction messaging was best received when described in the context of social or occasional use. Comparative messaging was appealing for harm reduction (ie, encouraging young adults who use cigarettes to quit smoking and use nicotine vapes), especially among LGBTQ+ participants. However, participants were concerned that comparative messages could unintentionally promote vaping among nicotine-naïve young adults. Qualitative participants preferred gain-framed efficacy messages that encouraged rather than demanded behavior change. Efficacy messages that emphasized “switching” were described as permissive for vaping, and participants were concerned that these may encourage sustained nicotine use. Some questioned whether vaping could effectively help young adults quit smoking. Survey results supported qualitative findings: messages with highest PME scores addressed toxic constituents, heart and lung disease, and cancer. Addiction messages were least effective. Among participants engaged in dual use, PME-smoking scores were higher when viewing candidate comparative messages than regulatory messages, but there were no significant differences between standard and comparative messages. The most effective candidate efficacy messages addressed quitting all smoking and vaping to reduce health risks. Messages that encouraged quitting smoking and switching to vapes were rated least effective.

**Conclusions:**

Comparative messaging was associated with higher PME-smoking among young adults engaged in dual use but did not consistently outperform standard messaging. Qualitative findings suggest that comparative framing may be misinterpreted as endorsing vaping as “safe” rather than “lower harm than cigarettes.” Further research is needed to examine potential unintended consequences of comparative messaging, including sustained nicotine use among young adults who smoke or normalization of vaping among nicotine-naïve young adults.

## Introduction

Concurrent use of nicotine vapes and combustible cigarettes (“dual use”) is a persistent and growing problem, particularly among young adults living in the United States [1-3]. Rates of dual use are higher among minoritized populations, including lesbian, gay, bisexual, transgender, and queer (LGBTQ+) young adults. In the United States, 5.5% of LGBTQ+ young adults report dual use, compared to 4.9% of their non-LGBTQ+ young adult counterparts [4]. Dual use reinforces nicotine dependence [5], is associated with continued tobacco use [6-8], and can lead to exclusive combustible smoking [9,10]. Identifying population-based strategies to reduce dual use among young adults, including LGBTQ+ populations, is necessary for advancing health equity.

Mass-reach health communication campaigns have been effective for preventing initiation [11-13] and progression of tobacco use [13,14] and reducing smoking [15-17]; however, no national US campaigns targeting young people have specifically addressed dual use. Existing campaigns (eg, The Real Cost and This Free Life) communicate health risks of a single product and aim to prevent initiation and progression [18,19], but these messages may not be effective in reducing use among young adults engaged in dual use. Specifically, communications focused on single-product health risks may trigger spillover effects, where users shift consumption to the nontargeted product rather than quitting all nicotine and tobacco. Additionally, standard risk messages (ie, those describing shared health risks of cigarettes and nicotine vapes) may not adequately reach this group, given heightened nicotine dependence associated with dual use and concomitant barriers to quitting all nicotine and tobacco. Additionally, very few mass-reach communication campaigns have been tailored to the LGBTQ+ young adult community, despite the evidence of increased mono- and dual-product use in this community.

In 2016, the US Food and Drug Administration (FDA) proposed a new nicotine-based framework for public health, in which they described products that deliver nicotine on a continuum of risk, based on toxicity and addictiveness [20]. Combustible products, including cigarettes, pose the highest risk, while other products, including nicotine vapes, demonstrate lower risk [21]. Consequently, the FDA Center for Tobacco Products and the US National Institutes of Health have prioritized research to develop effective messages about the health effects of nicotine vapes, including their harms compared to cigarettes [22]. Though not harmless, nicotine vapes pose fewer health risks than combustible cigarettes [21,23-25] and could present a lower-risk option for dual users not willing to quit nicotine entirely [26-28]. Evidence suggests that exposure to comparative risk communications (eg, “Vaping heats nicotine, resulting in lower levels of harmful chemicals than burned tobacco in cigarettes”) increases relative harm perceptions of cigarettes (vs nicotine vapes) [29], intentions to quit smoking [29], and intentions to switch to nicotine vapes [29-33] among adults who smoke. Studies present mixed findings on comparative messages and dual use: one study [32] found that comparative messages increased dual use interest among adults who smoke, while another [29] found that comparative messages did not increase dual use intentions. These studies did not assess responses to comparative messaging about nicotine vapes (vs cigarettes) among young adults, nor those engaged in dual use, specifically.

Comparative risk messages are expected to influence tobacco-related perceptions and intentions through a defined sequence of cognitive and affective responses. For example, messages that contrast the toxicant profile of cigarettes with that of nicotine vapes should heighten relative harm perceptions of cigarettes, which in turn should increase perceived message effectiveness (PME) for combustible cigarette smoking-related outcomes (PME-smoking) and strengthen intentions to quit smoking. At the same time, if comparative messages highlight the lower toxicant exposure associated with vaping, they may also elevate intentions to switch to exclusive nicotine vape use among individuals who smoke, particularly those engaged in dual use. This mechanism also creates the possibility of unintended vaping promotion among nonusers, leading to initiation. Comparative messages may also encourage uptake among mono-product users, resulting in sustained use among young adults who vape or dual use among young adults who smoke cigarettes. In contrast, standard messages that emphasize shared risks without explicit comparisons are expected to produce more uniform risk perceptions across products and fewer shifts in quit- or switch-related intentions. In this study, these hypothesized pathways informed the selection of outcomes, including PME-smoking, PME-vaping, and intentions to quit or switch.

A growing body of evidence suggests that comparative communications can effectively influence harm perceptions and behaviors among adults who smoke; however, little is known about how young adults—including those susceptible to or engaged in dual use—interpret and respond to such messages. The goal of this study was to develop candidate health communications that described either (1) shared risks of cigarettes and nicotine vapes (standard framing) or (2) comparative risks of cigarettes and nicotine vapes (comparative framing) with the aim of informing future campaigns to reduce dual use among young adults. Given documented disparities in dual use across subgroups of young adults, we assessed whether responses to standard and comparative messages differed by LGBTQ+ status. We also assessed if responses differed by current smoking and vaping status.

## Methods

We conducted a mixed methods formative study to develop and test candidate messages. The study included three sequential phases: (1) focus groups to inform message development, (2) in-depth interviews to qualitatively explore perceptions of thematic content (ie, topics, populations, and tone) applied in standard and comparative candidate messages, and (3) an online rating survey to quantitatively evaluate PME (ie, beliefs about how strongly a candidate message will influence behavior) and psychological reactance (ie, motivational resistance felt in response to a perceived threat to freedom of choice).

### Ethical Considerations

This study complied with the protection of human subjects in accordance with the ethical standards of the institutional review board and with the 1964 Helsinki Declaration and its later amendments. The study was approved by The Ohio State University Institutional Review Board (OSU-20427). Participants provided informed consent prior to participation.

### Developing Candidate Messages

#### Step 1: Focus Groups

Online focus groups were conducted with young adults who were currently engaged in or susceptible to dual use of cigarettes and nicotine vapes. Evidence indicates that individuals who exclusively use cigarettes or nicotine vapes often transition to later dual use, which is more commonly observed in younger adults [34]. Accordingly, we defined young adults who reported ever using both products and who were currently engaged in mono-product use as “susceptible” to future dual use. Participants were shown prompts about smoking cigarettes and vaping nicotine (Table S1 in Multimedia Appendix 1) and asked to evaluate semantic strategies (ie, language and tone) and thematic content (ie, message topics). Insights from focus groups informed the development of candidate standard (shared risk) and comparative (relative risk) messages. Given the rapid growth of nicotine vaping since 2017 [35], the peer-reviewed literature on vaping health effects is consistently updating; therefore, the research team continuously reviewed the scientific literature on health harms of nicotine vapes (and cigarettes) during the message development period (2021‐2022). Messages were reviewed by a mentorship team of leading tobacco and communication researchers. We also adapted regulatory warnings used by the FDA to compare outcomes for candidate messages.

#### Step 2: In-Depth Interviews to Explore Message Content

We conducted semistructured qualitative interviews with young adults (aged 18‐35 years) living in the United States who reported ever using cigarettes and nicotine vapes and who used at least 1 of those products in the past month. Eligibility criteria were informed by formative focus groups in which participants disagreed about the potential effects of comparative messaging on mono-product users (ie, concern that exposure to comparative messaging might support harm reduction among people who only smoke cigarettes but promote continued vaping among those who only vape nicotine). As such, we recruited young adults who reported ever using both products into our sample, including current mono-product users, so that we could further consider those concerns.

Our planned analytic sample size was 8, designed to maximize the information power of qualitative research [36,37], in which an initial sample of 6‐8 participants is recommended for interviews. We continued recruitment until we reached thematic saturation (n=13). Recruitment occurred via social media, flyers, emails to student organizations, a local LGBTQ+ health care organization, and word-of-mouth. Participants were screened for eligibility and consented into the study via phone. Consented participants were then sent a Qualtrics link to a baseline questionnaire about tobacco use and demographic characteristics. Interviews were conducted via Zoom (Zoom Video Communications), lasted about an hour, and participants received a US $30 electronic gift card.

An experienced facilitator used semistructured questions to guide the interviews. Participants were asked about their current and historical dual use prior to viewing 3 groups of candidate messages (12 standard, 12 comparative, and 12 efficacy messages; Table S2 in Multimedia Appendix 1). Messages were presented as text with no accompanying imagery. The presentation of the 3 groups and the order of candidate messages within groups were randomized. Participants were asked to read and respond to messages following a talk-aloud protocol in which they were prompted to identify the “best” or “worst” and “most effective” or “least effective” messages and to explain their reasoning.

All interviews were audio-recorded, transcribed, and checked by an independent transcriptionist for subsequent analysis. We applied thematic analysis to code and analyze interview data (Table 1). Transcripts were independently coded deductively and inductively by coding pairs (EEJ, GT, Elle Elson, and Sydney Galusha). Consensus meetings were held to review coding, reach agreement, and iteratively refine codes and definitions. Interrater reliability was assessed using percent agreement and Cohen κ. Although agreement between coders was high (97%‐99%), κ values were lower than expected (0.42‐0.49), likely due to low prevalence of some codes. To account for this limitation, we also calculated Gwet AC1 (0.96‐0.98), which indicated high interrater reliability independent of prevalence effects.

**Table 1.** Qualitative codebook for online interviews with young adults conducted between March and April 2022 examining standard risk, comparative risk, and efficacy messages regarding cigarette smoking and nicotine vaping.

Code	Definition	Statements, n (%)	Participants, n (%)
Message framing			
Short+simple	Discusses short+simple as an effective health communication tactic, either by saying a message would be better shorter or simpler or liking a message for that reason.	17 (2.62)	8 (61.54)
Facts/statistics=effective	Discusses facts and statistics as an effective health communication tactic.	17 (2.62)	7 (53.85)
Facts/statistics=ineffective	Discusses facts and statistics as NOT or LESS effective.	9 (1.39)	5 (38.46)
Constituents=effective	Discusses mentioning toxic constituents (formaldehyde, etc) as effective.	28 (4.31)	10 (76.92)
Constituents=ineffective	Discusses toxic constituents as NOT or LESS effective.	9 (1.39)	6 (46.15)
Fear=effective	Discusses “fear” as effective. May also use words like “scary” or “afraid.”	17 (2.62)	9 (69.23)
Fear=ineffective	Discusses “fear” as NOT or LESS effective.	2 (0.31)	2 (15.38)
Shock value=effective	Discusses “shock value” as effective. Participants may discuss being “surprised” by a message, making it effective.	4 (0.62)	1 (7.69)
Shock value=ineffective	Discusses “shock value” as NOT being effective.	2 (0.31)	1 (7.69)
Novelty=effective	Discusses “novelty” (ie, a new idea, or new information, “outside the box” thinking) as effective.	32 (4.93)	7 (53.85)
Novelty=ineffective	Discusses “novelty” (ie, a new idea, or new information, “outside the box” thinking) as ineffective.	7 (1.08)	3 (23.08)
Known truths=effective	Discusses how “knowing” that something is “true” (eg, smoking causes cancer) makes the message more effective (vs something novel or new or unheard of).	39 (6.01)	9 (69.23)
Known truths=ineffective	Discusses how “knowing” that something is “true” (eg, smoking causes cancer) makes the message NOT or LESS effective (vs something novel or new or unheard of).	24 (3.70)	9 (69.23)
Secondary effects=effective	Discusses how referencing health harms or other impacts of smoking or vaping (eg, financial) on family or friends or bystanders is effective.	2 (0.31)	1 (7.69)
Contextual factors			
Real-life context	Discusses how real-life context or current events (eg, opioid epidemic and COVID-19) influence their perceptions of the message.	4 (0.62)	4 (30.77)
Personal experience	Discusses how personal experience (either their own use or that of a peer/loved one) influences their perceptions of the message.	53 (8.17)	11 (84.62)
Prior quit attempts	Discusses how a history of prior quit attempts influences their perceptions of the message.	3 (0.46)	2 (15.38)
Audience	Discusses how audience (ie, whether target audience is smokers or vapers; established or not established users; and adolescents, young, or older adults) influences how effective or acceptable the message is.	74 (11.40)	13 (100)
Product and product use factors			
Comparative risk: vapes vs cigarettes	Discusses the comparative risk of vaping vs smoking combustible cigarettes (or the messaging around comparative risk).	7 (1.08)	5 (38.46)
Equivalent risk	Discusses how smoking and vaping have similar risks, or how referencing smoking or vaping in the same statement makes the risk seem equivalent.	13 (2)	7 (53.85)
Modified risk	Discusses nicotine vapes or vaping as a modified risk (healthier or lower harm) product than traditional combustible tobacco or smoking.	32 (4.93)	10 (76.92)
General health	Discusses general health when viewing the message, or generally discusses how the message evoked a thought about addiction, whether positive or negative.	46 (7.09)	13 (100)
General addictiveness	Discusses general addictiveness when viewing the message, or generally discusses how the message evoked a thought about addiction, whether positive or negative.	35 (5.39)	12 (92.31)
Harm reduction	Discusses using nicotine or tobacco as a harm reduction strategy (ie, instead of other drug use or to manage mental health or stress).	4 (0.62)	3 (23.08)
Switching	Discusses using “switch” language to describe vaping in lieu of smoking. May use terms including, but not limited to “turn to,” “change over,” “substitute,” “alternative,” or “use instead.”	9 (1.39)	7 (53.85)
Rate reduction	Discusses using information on how to decrease smoking on the path to quitting.	14 (2.16)	5 (38.46)
Off-ramp	Discusses how building an “off ramp” (ie, steps to quitting smoking, potentially including what to do once you know smoking is bad for you) is important content to include.	17 (2.62)	4 (30.77)
Language			
Permissive	Discusses how messaging or content is permissive (ie, makes vapes and/or cigarettes seem fine to use).	36 (5.55)	9 (69.23)
Tone	Discusses the tone of the message (eg, “preachy” and “friendly”).	27 (4.16)	8 (61.54)
Lay language	Discusses how using “lay” or “everyday” language is effective (eg, heart disease vs cardiovascular disease).	1 (0.15)	1 (7.69)
Scientific language	Discusses how using “scientific” or “professional” language is effective (eg, “cardiovascular disease” vs “heart disease”).	6 (0.92)	4 (30.77)
Specific language=effective	Discusses how using more specific language is effective (eg, “wheezing” vs “lung injury”).	22 (3.39)	10 (76.92)
Broad language=effective	Discusses how using broader language is effective (eg, “lung injury” vs “wheezing”).	7 (1.08)	3 (23.08)
Person-first language	Discusses using person-first language (eg, “people who smoke” or “using cigarettes” [implied person]) vs product-first language (eg, “smoker” or “vaper”).	2 (0.31)	1 (7.69)
General audience	Discusses using language to appeal to a general audience	1 (0.15)	1 (7.69)

#### Step 3: Online Rating Survey to Evaluate Message Perceptions

We conducted an online survey with 326 young adults (ages 18‐35 years) living in the United States. Participants were recruited through Prolific [38] using platform-based screeners to assess smoking and vaping status. Eligibility was restricted to individuals reporting ever using nicotine vapes and combustible cigarettes. Eligible participants who completed online informed consent were directed to a baseline survey administered via Qualtrics. Participants completed questions on nicotine and tobacco use and harm perceptions before viewing 5 randomly sampled candidate risk messages (of n=24 messages) and 3 randomly sampled candidate efficacy messages (of n=12 messages). Randomization was implemented using the Qualtrics Randomizer, which used a random selection algorithm without replacement within each session to ensure that every participant viewed 5 unique risk messages and 3 unique efficacy messages. To eliminate potential order effects, items were presented in a randomized sequence for each participant. Candidate messages were presented as text, with no accompanying images (Table S2 in Multimedia Appendix 1). After viewing each candidate message, participants completed questions assessing message perceptions and reactance. Participants were paid US $3.50 via Prolific.

To ensure data integrity, we cross-verified participants’ responses. In total, 37 respondents were excluded because their self-reported tobacco use history in the survey was incongruent with initial screening responses. After excluding 3 participants due to incomplete responses on key outcome variables or covariates, the final analytic sample included 286 US young adults who reported ever smoking and vaping.

### Measures

PME was assessed using the 3-item UNC Perceived Message Effectiveness Scale (original α=.90), which assesses effects perceptions or beliefs about a message’s potential to change behavior [39]. Meta-analyses demonstrate that PME is an empirically meaningful predictor of quit intentions and cessation behavior [40]. PME-smoking and PME-vaping measures included 3 items on a 5-point Likert scale (1=strongly disagree and 5=strongly agree), assessing whether message exposure discouraged use, made smoking or vaping seem unpleasant, and made participants concerned about health effects of smoking or vaping (PME-smoking α=.93; PME-vaping α=.96). Scores were summed and averaged to create 2 unique variables assessing each candidate message’s potential to influence smoking behavior (PME-smoking) and vaping behavior (PME-vaping). As part of the paper revision process, we reaudited the PME-smoking scale to ensure accurate scoring and item handling; the reliability estimate reported here reflects the corrected and final scoring approach.

Efficacy messages were assessed by their perceived effectiveness (PE) for motivating people to quit cigarettes and nicotine vapes on a 5-point Likert scale (1=strongly disagree and 5=strongly agree) with the statement: “This message will motivate people to quit all e-cigarette and combustible cigarette products” (PE-quit). We also assessed PE for motivating people to switch from cigarettes to nicotine vapes using the same scale and the statement: “This message will motivate people who smoke cigarettes to switch completely to vaping e-cigarettes” (PE-switch).

Psychological reactance measures a person’s negative reactions to a message, which may preclude counterarguing [41]. We measured reactance to candidate risk and efficacy messages with 3 items: “This message is trying to manipulate me,” “This message annoys me,” and “The health effect of this message is overblown,” scored on a 5-point response scale ranging from 1=strongly disagree to 5=strongly agree (original α=.75) [41]. Scores were summed and averaged for a total reactance score variable where higher scores equal higher psychological reactance to the message (α=.83).

### Statistical Analysis

To estimate message-level effects while accounting for the incomplete-block design, we used cross-classified linear mixed-effects models with random intercepts for participants and messages. This approach modeled repeated observations nested within participants and candidate messages.

Models were estimated separately for PME (smoking and vaping), PE of efficacy messages (quit and switch), and psychological reactance. For each outcome, we first estimated an unconditional (intercept-only) model and used likelihood ratio (LR) tests to evaluate the necessity of the multilevel structure. Model specification was iteratively evaluated using LR tests. Random slopes did not significantly improve fit for any model and were excluded to maintain parsimony. For the PME-smoking and vaping models, demographic covariates failed to improve fit, justifying the use of intercept-only structures. Conversely, model fit was significantly improved by adding sex-at-birth to the PE-quit and PE-switch models, and a binary age indicator (18‐25 vs 26‐35 years) to both psychological reactance models. From these final models, we derived message-specific adjusted means and 95% CIs via partial pooling to stabilize individual message estimates. Finally, to control the inflation of type I error across multiple outcomes, we applied Bonferroni corrections separately within each experiment based on the number of primary dependent variables evaluated (3 outcomes per experiment, Bonferroni-corrected α=0.0167).

We then assessed systematic differences in message framing effects by LGBTQ+ status and smoking or vaping status using linear regressions with robust SEs. To examine whether framing effects varied by group, we included product interaction terms in all models, regardless of main effect significance. If interactions were not statistically significant (*P*<.05), we removed the interaction terms and presented main effect models only. A Bonferroni-corrected α of 0.004 was applied to control for the family-wise error rate across the 12 primary models (6 outcomes×2 moderators). Significant interactions (*P*<.05) were decomposed by analyzing Bonferroni-corrected simple effects of message framing within each level of LGBTQ+ (2 levels; Bonferroni-corrected α=0.025) and smoking or vaping status (4 levels; Bonferroni-corrected α=0.0125). When simple effects were significant, we calculated predictive margins to generate means and 95% CIs and conducted post-hoc pairwise comparisons within stratified groups to identify differences (Sidak-adjusted *P*=.05). All analyses were conducted in Stata SE (version 18.0; StataCorp).

## Results

### Step 1: Focus Groups

All participants (N=12) reported past 30-day nicotine vaping, and 11 reported past 30-day cigarette use. They were, on average, 23.75 (SD 4.07) years of age, and 7 identified as LGBTQ+.

#### Language

Generally, participants preferred prompts that applied person-first language (eg, “people who smoke” vs “smokers”) and were less enthusiastic about prompts referring to specific subgroups of “young people” or “young adults.” Participants preferred “everyday” language to describe electronic nicotine products, including “vaping” or “vapes” (vs e-cigarettes) but noted that messages should specify “nicotine vaping / vapes” to avoid confusion with nonnicotine vape products (ie, cannabis, caffeine, and herbal).

#### Content

Overall, standard and comparative message prompts describing short- and long-term physical health harms and toxic constituents were perceived as effective and memorable. Those describing nicotine addiction were perceived as least effective. Participants described harm messages that induced fear as attention-getting and effective. Emerging young adults (aged 18‐25 years) described how prior exposure to antitobacco campaigns during K-12 education (eg, Drug Abuse Resistance Education) attenuated the effectiveness of messages describing chronic tobacco-related diseases (eg, cancer and lung disease). As such, emerging young adults found more appeal in message prompts highlighting short-term health harms (eg, effects of nicotine on sleep) and lesser-known toxic constituents (eg, formaldehyde).

#### Comparative Risk Messaging

Participants held diverse opinions about the salience of comparative risk messages for young people susceptible to dual use. Some appreciated that comparative messages might encourage young adults using combustible cigarettes (either mono-product or dual use) to move down the tobacco harm continuum by vaping only. Others were skeptical of comparative messages and were concerned that they might encourage more frequent vape use among young people who only vape.

#### Efficacy Messaging

Participants described how health communications that solely provided information about harms were insufficient. In addition to providing quit resources for individuals interested in quitting, participants suggested that messages include information about cessation and harm reduction strategies, including encouraging statements and “how to” tips (which they described as “off-ramp” messaging). In response, we developed a series of candidate messages addressing behavioral efficacy, which we explored in qualitative interviews (Table S2 in Multimedia Appendix 1).

### Step 2: In-Depth Interviews to Explore Message Content

#### Sample Characteristics

In total, 13 participants completed interviews, 7 identified as cisgender male, 5 were White, and 9 identified as LGBTQ+. Their average age was 24.69 (SD 3.71) years. Of participants, 12 used cigarettes, and 11 vaped nicotine during the month of their interview (Table 2).

**Table 2.** Demographic characteristics of young adult participants of online interviews conducted between March and April 2022 examining standard risk, comparative risk, and efficacy messages regarding cigarette smoking and nicotine vaping.

	Values, n (%)
Gender	
Cisgender female	4 (30.77)
Cisgender male	7 (53.85)
Other	2 (15.38)
Sexual orientation	
Heterosexual	4 (30.77)
Lesbian or gay	5 (38.46)
Pansexual	2 (15.38)
Other	2 (15.38)
Race	
Black or African American	5 (38.46)
White	5 (38.46)
Other people of color	3 (23.08)
Age (years)	
18-25	6 (46.15)
26-35	7 (53.85)
Use in the last month	
Cigarettes	12 (92.31)
Nicotine vapes	11 (84.62)
Cigars	5 (38.46)
Little cigars	4 (30.77)
Hookah	1 (7.69)

#### Theme 1: Messages Describing Novel Content Were Perceived Effective, While “Known Truths” Had Mixed Response

Participants, including a greater proportion of non-LGBTQ+ young adults, felt that novelty was important in candidate messages. When asked what information we should highlight, one participant responded:

Lesser-known information that I’ve never even heard before. I think that would actually get a lot of attention.

[18‐25 years, non-LGBTQ+, current dual use]

Participants considered messages describing toxic constituents and DNA damage novel and explained that, because the public is less aware of these health effects, it increases “shock value”:

It’s a consequence you don’t hear about as much about ... the metals part of it. And then also, like, arsenic and lead sound just really dangerous.

[18‐25 years, non-LGBTQ+, current dual use]

Some participants felt messages required novel information to engage their attention; otherwise, messages would be redundant with prior K-12 antitobacco education.

I feel like since I had DARE, it hasn’t come out that there’s like these new harms of smoking nicotine cigarettes that’s like “Oh, no one knew this.” We get it. It’s horrible for you in every way, so while it’s nice that the other messages, like, say specific things, I think I already know what the harm is.

[18‐25 years, non-LGBTQ+, current cigarette use]

In contrast, LGBTQ+ participants responded positively to messages describing scientifically established health harms (eg, cancer). Said one participant, “Heart disease is so common ... highlighting physical health risks as the danger [is effective], rather than, ‘Oh, it’s just bad for you’” (18‐25 years, LGBTQ+, current nicotine vape use). LGBTQ+ participants also described how known physical harm messages that induced fear were more attention-getting: “Cancer is one of those statement shocker words of ‘Oh, I could get this?’ I just think the word ‘cancer,’ to see it in any kind of ad ... you stop for a second and you look” (26‐35 years, LGBTQ+, current dual use).

Responses to messages on nicotine addiction were mixed. Messages stating that “nicotine is addictive” were generally considered ineffective: “There’s no point, everyone knows that” (18‐25 years, non-LGBTQ+, current dual use). However, messages presenting novel information about nicotine addiction (eg, in the context of social or occasional use) were viewed as effective. Responding to the message, “Even occasional smoking and vaping can lead to nicotine addiction,” one participant noted:

If I had heard that earlier on, with maybe some more force, I would have probably been a lot more abstinent. Because my whole thinking was like, “Well, I’m not like everyone else, I won’t get addicted. I’m too good for that.” So, it’s finding a way to like, broaden it and make it feel like ... you’re not special. You’ll get addicted. Everyone gets affected by nicotine the same way.

[18‐25 years, non-LGBTQ+, current dual use]

#### Theme 2: Tensions Exist When Communicating the Harm Minimization Potential of Vaping

Comparative candidate messages were appealing for their potential for harm minimization. Several participants, including a greater proportion of LGBTQ+ young adults, remarked that comparative messages were effective as a mechanism to reduce the harm of combustible smoking:

There will be some harm reduction if people that smoke cigarettes daily can become more casual vapers. Yeah, so I like [this message].

[18‐25 years, LGBTQ+, current nicotine vape use]

Other participants interpreted comparative messages to mean that vaping is an effective strategy for quitting smoking:

The reason I like [the message] is, vaping is a way of trying to quit smoking, but it does not mean it’s not harmful at all. So, you have to pass across the message that it is still harmful, but it has a reduced risk from smoking. So, when you’re comfortable and try and quit, vaping will help you.

[18‐25 years, LGBTQ+, current dual use]

Efficacy messages emphasizing “switching” were viewed as permissive for vaping. Participants were concerned that efficacy messages directing users to “switch” to vapes might encourage continued nicotine use instead of quitting. In describing this concern, some participants implicitly equated smoking and vaping harms:

I think [this message] is another excuse to keep doing what you’ve been doing, right? You just quit smoking and just go to vaping. I think that isn’t really much different ... I think if you want to quit, you should quit.

[26‐35 years, LGBTQ+, current dual use]

Other participants questioned whether vaping was effective for helping smokers eventually quit all nicotine: “... is there research on—like do people that go from smoking to nicotine [vaping], do they ever end up quitting?” (18‐25 years, LGBTQ+, current nicotine vape use).

Tone was important to young adults when assessing the effectiveness of harm minimization and efficacy messages. Most participants, but especially those aged 18‐25 years, preferred when efficacy messages encouraged rather than demanded behavior change. Leading questions (eg, “Not ready to quit completely?”) and empathetic opening statements were described as effective strategies for engaging participants in behavior change: “The way [the messages are] phrased makes it seem that it’s like, it’s my choice to do it, rather than being told to do something” (18‐25 years, LGBTQ+, current nicotine vape use).

### Step 3: Online Rating Survey to Evaluate Message Perceptions

#### Sample Characteristics

Most participants self-identified as White (186/286, 65.03%), 39.16% (112/286) were aged 18‐25 years, 54.55% (156/286) were assigned female at birth, and 51.75% (148/286) identified as LGBTQ+ (Table 3). Of all participants, 30.07% (86/286) reported current dual use, 19.93% (57/286) smoked, 31.47% (90/286) vaped, and 18.53% (53/286) did not currently smoke or vape. LGBTQ+ participants were younger than non-LGBTQ+ participants (*P*=.007; LGBTQ+: 69/148, 46.62% vs non-LGBTQ: 43/138, 31.16% in the 18‐25 age group). More LGBTQ+ participants were assigned female at birth (*P*=.001; LGBTQ+: 95/148, 64.19% vs non-LGBTQ+: 61/138, 44.2%).

**Table 3.** Sample demographic characteristics for an online survey sample of US young adults (aged 18‐35 years) reporting ever using both cigarettes and nicotine vapes, conducted between February and April 2022.

	Full sample (N=286), n (%)	LGBTQ+^a^ (n=148), n (%)	Non-LGBTQ+ (n=138), n (%)	*P* value	No current use (n=53), n (%)	Current vaping (n=90), n (%)	Current smoking (n=57), n (%)	Current dual use (n=86), n (%)	*P* value
Age group (years)				.007					<.001
18‐25	112 (39.16)	69 (46.62)	43 (31.16)		17 (32.08)	48 (53.33)	10 (17.54)	37 (43.02)	
26‐35	174 (60.84)	79 (53.38)	95 (68.84)		36 (67.92)	42 (46.67)	47 (82.46)	49 (56.98)	
Sex assigned at birth				.001					.61
Female	156 (54.55)	95 (64.19)	61 (44.20)		25 (47.17)	52 (57.78)	33 (57.89)	46 (53.49)	
Male	130 (45.55)	53 (35.81)	77 (55.80)		28 (52.83)	38 (42.22)	24 (42.11)	40 (46.51)	
Hispanic or Latinx	28 (9.79)	14 (9.46)	14 (10.14)	.85	7 (13.21)	6 (6.67)	7 (12.28)	8 (9.30)	.55
Race				.35					.09
American Indian or Alaska Native	2 (0.70)	2 (1.35)	0 (0)		0 (0)	0 (0)	0 (0)	2 (2.33)	
Asian or Pacific Islander	14 (4.90)	8 (5.41)	6 (4.35)		4 (7.55)	4 (4.44)	1 (1.75)	5 (5.81)	
Black or African American	32 (11.19)	13 (8.78)	19 (13.77)		6 (11.32)	4 (4.44)	9 (15.79)	13 (15.12)	
White	186 (65.03)	102 (68.92)	84 (60.87)		36 (67.92)	58 (64.44)	38 (66.67)	54 (62.79)	
Multiracial	37 (12.94)	16 (10.81)	21 (15.22)		3 (5.66)	19 (21.11)	5 (8.77)	10 (11.63)	
Another race or ethnicity	15 (5.24)	7 (4.73)	8 (5.80)		4 (7.55)	5 (5.56)	4 (7.02)	2 (2.33)	
Education level				.51					.19
High school diploma or GED^b^ or lower	61 (21.33)	32 (21.62)	29 (21.01)		12 (22.64)	15 (16.67)	15 (26.32)	20 (22.09)	
Vocational or associate degree	35 (12.24)	16 (10.81)	19 (13.77)		9 (16.98)	13 (14.44)	6 (10.53)	7 (8.14)	
Some college (not graduated)	88 (30.77)	52 (35.14)	36 (26.09)		11 (20.75)	31 (34.44)	19 (33.33)	27 (31.40)	
Bachelor degree	89 (31.12)	42 (28.38)	47 (34.06)		15 (28.30)	30 (33.33)	16 (28.07)	28 (32.56)	
Master degree or higher	13 (4.55)	6 (4.05)	7 (5.07)		6 (11.32)	1 (1.11)	1 (1.75)	5 (5.81)	
Use status				.95	—^c^	—	—	—	—
No current use	53 (18.53)	29 (19.59)	24 (17.39)						
Current vaping only	90 (31.47)	47 (31.33)	43 (31.16)						
Current smoking only	57 (19.93)	28 (18.92)	29 (21.01)						
Current dual use	86 (30.07)	44 (30.43)	42 (30.43)						

a LGBTQ+: lesbian, gay, bisexual, transgender, and queer.

b GED: General Educational Development test.

c Not applicable.

#### Risk Messages

##### Overview

Table 4 presents parameter estimates and variance components for the cross-classified mixed models. Across all 3 outcomes, LR tests comparing the cross-classified models to standard ordinary least squares linear regressions were highly significant (*P*’s<.001). Participant-level variance accounted for the majority across outcomes (range 40.9%‐47.5%), while 7%‐16.2% of the variance was due to the specific messages viewed.

##### Mean PME Scores

###### Overview

Risk message-specific marginal means are presented in Table 5 in order of most to least effective for PME-smoking within standard and comparative message framing categories.

**Table 4.** Cross-classified linear mixed models for standard and comparative risk candidate messages about cigarette smoking and nicotine vaping tested in an online survey of US young adults between February and April 2022 (N=286)^a,b^.

Parameter	PME^c^-vaping, estimate (95% CI)	PME-smoking, estimate (95% CI)	Reactance, estimate (95% CI)
Fixed effects			
Intercept	2.87 (2.62-3.13)^d^	3.57 (3.38-3.77)^d^	2.05 (1.86-2.24)^d^
18‐25 years	—^e^	—	0.24 (0.05-0.43)^f^
Random effects (variance)			
Participant σ2	0.83 (0.68-1.02)	0.79 (0.65-0.96)	0.52 (0.42-0.64)
Message σ2	0.32 (0.18-0.58)	0.16 (0.08-0.29)	0.09 (0.05-0.17)
Residual σ2	0.82 (0.75-0.89)	0.72 (0.66-0.78)	0.66 (0.61-0.72)

a A total of 1430 observations across all models. Fixed effects display unstandardized coefficients. Random effects display variance components. A Bonferroni correction was applied for 3 primary outcomes (α=.0167).

b Model fit indices: PME-vaping: log-likelihood=−2181.43; likelihood ratio (LR) test X2 vs ordinary least squares (OLS)=633.64 (*P*<.001); PME-smoking: log-likelihood=−2091.70; LR test X2 vs OLS=602.76 (*P*<.001); reactance: log-likelihood=−1983.02; LR test X2 vs OLS=434.86 (*P*<.001).

c PME: perceived message effectiveness.

d *P*<.001.

e Not available.

f *Indicates statistical significance (P*<.0167.)

**Table 5.** Mean perceived message effectiveness and psychological reactance scores for standard and comparative risk candidate messages about cigarette smoking and nicotine vaping tested in an online survey of US young adults between February and April 2022 (N=286).

ID#	Type	Message	PME^a^-smoking, mean (95% CI)	PME-vaping, mean (95% CI)	Reactance, mean (95% CI)
9	Candidate standard	Smoking cigarettes and vaping nicotine increases your risk for heart disease.	4.06 (3.83-4.30)	3.64 (3.39-3.90)	1.78 (1.56-1.99)
11	Candidate standard	Wheezing lately? Smoking cigarettes and vaping nicotine increases your risk for lung problems.	4.00 (3.77-4.24)	3.49 (3.23-3.75)	1.86 (1.65-2.08)
3	Candidate standard	Cigarettes and some nicotine vapes contain formaldehyde, a chemical that causes cancer.	3.99 (3.76-4.22)	3.66 (3.41-3.91)	1.84 (1.63-2.05)
8	Candidate standard	Smoking cigarettes and vaping nicotine damages your DNA and increases your risk for cancer.	3.79 (3.55-4.02)	3.49 (3.23-3.74)	2.05 (1.84-2.27)
5	Candidate standard	Using cigarettes and nicotine vapes exposes you to heavy metals—like arsenic and lead.	3.76 (3.52-3.99)	3.57 (3.31-3.82)	1.90 (1.68-2.11)
12	Candidate standard	Can’t find your keys? Nicotine in cigarettes and vapes can harm your brain and lead to memory loss.	3.56 (3.33-3.79)	3.25 (3.00-3.50)	2.47 (2.26-2.68)
10	Candidate standard	Can’t sleep? Nicotine in cigarettes and vapes can lead to sleep problems, including insomnia.	3.49 (3.23-3.74)	3.36 (3.08-3.64)	1.95 (1.72-2.18)
2	Candidate standard	Most people think smoking cigarettes and vaping nicotine are harmful to your health. They’re right.	3.44 (3.20-3.67)	3.26 (3.00-3.52)	1.95 (1.74-2.17)
4	Candidate standard	Nicotine doesn’t cause cancer, but toxic chemicals in e-liquid and tobacco can.	3.42 (3.19-3.65)	3.15 (2.89-3.40)	2.10 (1.88-2.31)
6	Candidate standard	Even occasional smoking and vaping can lead to nicotine addiction.	3.30 (3.06-3.54)	2.91 (2.64-3.17)	1.86 (1.65-2.08)
7	Regulatory standard	Cigarettes and vapes contain nicotine. Nicotine is an addictive chemical.	3.10 (2.88-3.32)	2.96 (2.72-3.19)	1.60 (1.40-1.80)
15	Candidate comparative	Cigarettes contain more cancer-causing chemicals—like formaldehyde—than nicotine vapes.	4.05 (3.81-4.30)	2.25 (1.98-2.52)	1.91 (1.69-2.14)
16	Candidate comparative	Many people think nicotine in vapes and cigarettes causes cancer. Truth is, toxic chemicals in burning cigarettes are more likely to cause cancer.	3.90 (3.66-4.14)	2.47 (2.20-2.73)	1.88 (1.66-2.10)
21	Candidate comparative	People who smoke cigarettes and vape nicotine have a higher risk of developing heart disease than those who only vape.	3.89 (3.66-4.13)	2.79 (2.53-3.05)	1.93 (1.72-2.15)
23	Candidate comparative	Wheezing much? People who smoke cigarettes and vape nicotine report more lung problems than those who only vape.	3.84 (3.59-4.09)	2.93 (2.66-3.20)	2.12 (1.89-2.35)
17	Candidate comparative	Using cigarettes and nicotine vapes exposes you to more heavy metals—like arsenic and lead—than if you only vape.	3.76 (3.53-4.00)	3.00 (2.74-3.26)	2.03 (1.81-2.24)
20	Candidate comparative	Smoking cigarettes can increase your risk for lung, head, and neck cancer. Using nicotine vapes only may decrease your cancer risk.	3.74 (3.50-3.99)	2.20 (1.93-2.47)	2.31 (2.09-2.54)
13	Candidate comparative	Vaping nicotine is harmful to your health, but it is less harmful than smoking.	3.62 (3.39-3.85)	2.33 (2.08-2.58)	2.14 (1.93-2.34)
14	Candidate comparative	Many people think vaping nicotine is just as harmful as smoking cigarettes. Truth is, nicotine vapes are less harmful than cigarettes.	3.45 (3.21-3.69)	1.87 (1.61-2.13)	2.13 (1.91-2.35)
19	Candidate comparative	Nicotine vapes are addictive, but people who vape and smoke tobacco are 7 times more likely to be addicted to nicotine than those who only vape.	3.44 (3.20-3.67)	2.66 (2.41-2.92)	1.93 (1.72-2.14)
22	Candidate comparative	Can’t sleep? People who vape and smoke report more sleep problems than those who only vape.	3.42 (3.19-3.65)	2.44 (2.18-2.69)	2.27 (2.06-2.48)
18	Candidate comparative	Vaping heats nicotine, resulting in lower levels of harmful chemicals than burned tobacco in cigarettes.	3.41 (3.17-3.65)	2.12 (1.86-2.38)	1.98 (1.76-2.19)
1	Regulatory comparative	Nicotine vapes are not a safe alternative to cigarettes.	2.69 (2.45-2.93)	3.26 (3.00-3.52)	2.08 (1.86-2.30)
24	Candidate comparative	Can’t find your keys? Research suggests that vaping nicotine can improve attention and memory during smoking abstinence.	2.60 (2.36-2.83)	1.92 (1.67-2.18)	3.06 (2.84-3.27)

a PME: perceived message effectiveness.

###### Standard Risk Messages

Most standard candidate messages scored PMEs above the midpoint score (>3) for smoking and vaping. Those with the highest PMEs addressed the risk of heart disease (ID 9), lung disease (ID 11), cancer (IDs 3 and 8), and toxic constituents, including formaldehyde (ID 3) and heavy metals (ID 5). Two standard candidate messages had lower PMEs for smoking and vaping; both messages addressed the addictive potential of nicotine (IDs 6 and 7).

###### Comparative Risk Messages

Like standard candidate messages, most comparative candidate messages scored PMEs for smoking behavior >3. The comparative candidate messages with the highest PME-smoking scores addressed how smoking increases cancer risk more than vaping (IDs 15 and 16), heart disease (ID 21), lung disease (ID 23), and heavy metals (ID 17). With respect to PME-vaping scores, comparative candidate messages were less effective. Only one message scored above 3 for PME-vaping (ID 1).

### Mean Psychological Reactance Scores

Almost all standard and comparative candidate messages scored below the midpoint for psychological reactance, indicating that participants did not respond negatively to the messages (Table 5). Messages with the highest reactance discussed effects of smoking and vaping on memory (IDs 12 and 24).

### Effect of Risk Message Framing on Outcomes

#### Overview

Table 6 presents effects of risk message framing on PME and reactance. Preliminary analyses indicated that LGBTQ+ status did not produce significant interaction effects across any outcome (results not shown).

**Table 6.** Effect of risk message framing on perceived message effectiveness (PME) and reactance: findings from an online survey of US young adults reporting ever using both cigarettes and nicotine vapes conducted between February and April 2022 (N=286)^a^.

	Mean (95% CI)	*P* value (vs reference)^b^	*P* value (vs regulatory comparative message)^b^	*P* value (vs standard candidate message)^b^
Interaction effects of message framing on PME-smoking, by current use status^c^				
PME-smoking—current dual use				
Regulatory standard message (reference)	2.85 (2.39-3.32)	—^d^	—	—
Regulatory comparative message	1.81 (1.41-2.20)	*<.001* ^e^	—	—
Standard candidate messages	3.38 (3.13-3.63)	.22	*<.001*	—
Comparative candidate messages	3.56 (3.35-3.77)	*.03*	*<.001*	.43
Main effects of message framing on PME-vaping				
Regulatory standard message (reference)	2.99 (2.66-3.32)	—	—	—
Regulatory comparative message	3.38 (3.05-3.72)	.43	—	—
Standard candidate messages	3.39 (3.25-3.54)	.10	>.99	—
Comparative candidate messages	2.39 (2.26-2.51)	*.002*	*<.001*	*<.001*
Main effects of message framing on reactance				
Regulatory standard message (reference)	1.68 (1.48-1.87)	—	—	—
Regulatory comparative message	2.10 (1.82-2.39)	.08	—	—
Standard candidate messages	2.12 (2.00-2.23)	*<.001*	>.99	—
Comparative candidate messages	2.32 (2.20-2.44)	*<.001*	.63	*.02*

a A total of 286 young adults viewed 5 randomly selected textual health messages (of N=24 messages) with differing message framing. Linear regression models with generalized estimating equations were used to model differences in outcome variable means by message risk framing (regulatory standard, regulatory comparative, standard candidate, comparative candidate). All models controlled for baseline differences. When no interactions were identified, statistically significant main effects models without interaction terms are reported.

b *P* values were calculated using Wald tests. We assessed statistical significance for overall main effects (6 outcomes×2 moderators) using a Bonferroni-corrected α=0.004 to control for familywise error rate. When there were significant main effects, post-hoc pairwise comparisons were conducted using Sidak adjustment.

c Significant interactions between message type and nicotine vape or cigarette use status were identified and decomposed via simple main effects within each level of the moderator. To minimize family-wise error at the decomposition stage, we evaluated simple main effects against a Bonferroni-corrected α=0.0125 (adjusting for the 4 moderator levels); only strata reaching this significance threshold are displayed. Post-hoc pairwise comparisons were conducted using Sidak adjustment. All participants reported ever using both cigarettes and nicotine vapes; participants were categorized by self-reported current use of cigarettes and nicotine vapes in the past 30 days as follows: current nonuse of cigarettes or nicotine vapes, current nicotine vape vaping only, current cigarette smoking only, or current dual use of cigarettes and nicotine vapes.

d Not available.

e Significant differences indicated in italics format (*P*<.05).

#### PME-Smoking

We observed a significant interaction between message framing and current nicotine or tobacco use status (*P*=.003). Simple effects decomposition revealed that framing significantly influenced PME-smoking scores among young adults reporting current dual use (*P*=.003). Within this group, Sidak-adjusted pairwise comparisons showed that those viewing comparative candidate messages (mean 3.56, 95% CI 3.36‐3.77) reported higher PME-smoking than those viewing the regulatory standard (mean 2.85, 95% CI 2.39-3.32; *P*=.03) or regulatory comparative message (mean 1.81, 95% CI 1.41-2.20; *P*<.001). Standard candidate messages (mean 3.38, 95% CI 3.13-3.63) were rated significantly more effective than regulatory comparative messages (*P*<.001).

#### PME-Vaping

We found no significant interaction effects for PME-vaping. Main effects models revealed that message framing was associated with PME-vaping (*P*<.001 at the Bonferroni-corrected α=0.004). Sidak-adjusted pairwise comparisons showed that participants rated comparative candidate messages (mean 2.39, 95% CI 2.26-2.51) as less effective for influencing vaping behavior than standard candidate messages (mean 3.39, 95% CI 3.25-3.54; *P*<.001), regulatory comparative messages (mean 3.38, 95% CI 3.05-3.72; *P*<.001), or regulatory standard messages (mean 2.99, 95% CI 2.66-3.32; *P*=.002).

#### Reactance

We found no significant interaction effects for reactance. Main effects models revealed that message framing was associated with reactance (*P*<.001 at the Bonferroni-corrected α=0.004). Sidak-adjusted pairwise comparisons indicated that participants rated regulatory standard messages (mean 1.68, 95% CI 1.48-1.87) lower for reactance than standard candidate (mean 2.12, 95% CI 2.00-2.23; *P*<.001) and comparative candidate (mean 2.32, 95% CI 2.20-2.44; *P*<.001) messages. Standard candidate messages were rated lower for reactance than comparative candidate messages (*P*=.02).

### Efficacy Messages

#### Overview

Table 7 presents parameter estimates and variance components for the cross-classified mixed models. Across all 3 outcomes, LR tests comparing the cross-classified models to standard ordinary least squares linear regressions were significant (*P*’s<.001). Participant-level variance accounted for the majority across outcomes (range 26.3%‐49.7%), while 1.4%‐24.6% of the variance was due to the specific messages viewed. Participants aged 18‐25 years experienced significantly higher reactance to the messages than those aged 26‐35 years (B=0.30, 95% CI 0.08-0.52; *P*=.008).

**Table 7.** Cross-classified linear mixed models for standard and comparative risk candidate messages about cigarette smoking and nicotine vaping tested in an online survey of US young adults between February and April 2022 (N=286)^a,b^.

Parameter	PE^c^-quit, estimate (95% CI)	PE-switch, estimate (95% CI)	Reactance, estimate (95% CI)
Fixed effects			
Intercept	2.59 (2.25 to 2.94)^d^	2.94 (2.57 to 3.32)^d^	1.83 (1.64 to 2.02)^d^
Sex at birth: female	−0.24 (−0.44 to −0.03)	−0.22 (−0.43 to −0.02)	—^e^
Young age category (group 1)	—	—	0.30 (0.08 to 0.52)^f^
Random effects (variance)			
Participant σ2	0.46 (0.34 to 0.61)	0.44 (0.33 to 0.60)	0.64 (0.51 to 0.80)
Message σ2	0.30 (0.13 to 0.71)	0.37 (0.16 to 0.85)	0.02 (0.01 to 0.07)
Residual σ2	0.98 (0.87 to 1.10)	0.98 (0.88 to 1.11)	0.63 (0.56 to 0.71)

a A total of 858 observations across all models. Fixed effects display unstandardized coefficients. Random effects display variance components. A Bonferroni correction was applied for 3 primary outcomes (α=.0167).

b Model fit indices: PE-quit: log-likelihood=−1350.9; likelihood ratio (LR) test X2 vs ordinary least squares (OLS)=197.03 (*P*<.001); PE-switch: log-likelihood=−1351; LR test X2 vs OLS=219.26 (*P*<.001); reactance: log-likelihood=−1226.4; LR test X2 vs OLS=201.47 (*P*<.001).

c PE: perceived effectiveness.

d *P*<.001.

e Not available.

f *Indicates statistical significance (P*<.0167).

#### Mean PE Scores

Efficacy message-specific marginal means are presented in Table 8 in order of most to least effective for PE-smoking. Efficacy messages that directed viewers to quit all smoking and vaping to reduce health risks (IDs Q1 and Q3) and exposures (Q2) were rated most effective for motivating quitting behaviors, but least effective for motivating people who smoke to switch to vaping only (Table 5). Candidate messages that described how switching to vaping only may reduce health risks (IDs S1, S2, and C2) were rated most effective for motivating switch behaviors.

**Table 8.** Mean perceived effectiveness of candidate smoking and vaping messages to motivate quitting and switching behaviors and psychological reactance scores for candidate messages tested in an online rating survey of US young adults between February and April 2022 (N=286).

	Message	Effectiveness for motivating quitting smoking, mean (95% CI)	Effectiveness for motivating switching to vapes only, mean (95% CI)	Psychological reactance, mean (95% CI)
Q2	Quit all smoking and vaping to reduce your cancer risk.	3.51 (3.25-3.78)	2.21 (1.94-2.47)	1.74 (1.57-1.91)
Q3	Quit smoking and vaping completely to reduce your exposure to toxic chemicals.	3.28 (3.03-3.53)	2.34 (2.09-2.59)	1.77 (1.60-1.93)
Q1	Quit all smoking and vaping to reduce serious risks to your health.	3.06 (2.78-3.33)	2.26 (1.98-2.54)	1.86 (1.69-2.04)
Q4	Ready to quit smoking and vaping completely? Manage your cravings with nicotine replacement therapy.	3.04 (2.76-3.32)	2.34 (2.06-2.63)	1.77 (1.59-1.95)
C4	If you’re not ready to quit all nicotine, using nicotine replacement therapy can help you manage cravings and quit smoking.	2.92 (2.68-3.16)	2.61 (2.37-2.86)	1.64 (1.48-1.81)
S4	Manage your smoking cravings with nicotine replacement therapy.	2.77 (2.53-3.01)	2.54 (2.30-2.78)	1.72 (1.56-1.88)
S1	Switching completely from smoking to vaping nicotine can reduce serious risks to your health.	2.29 (2.04-2.54)	3.59 (3.34-3.84)	2.02 (1.86-2.19)
C2	If you’re not ready to quit all nicotine, switching completely to vaping may reduce your cancer risk.	2.15 (1.89-2.41)	3.89 (3.63-4.15)	1.83 (1.66-2.00)
S3	Switching from smoking to vaping only can reduce your exposure to toxic chemicals.	2.12 (1.86-2.38)	3.07 (2.81-3.33)	1.89 (1.72-2.06)
S2	Switching completely from smoking to vaping nicotine can reduce your cancer risk.	2.10 (1.85-2.35)	3.51 (3.26-3.76)	1.91 (1.74-2.07)
C3	Start quitting smoking by switching to nicotine vapes only.	1.96 (1.72-2.20)	3.47 (3.23-3.70)	1.97 (1.81-2.13)
C1	Not ready to quit completely? Quit smoking all tobacco, and use nicotine vapes only to reduce serious risks to your health.	1.94 (1.70-2.19)	3.46 (3.22-3.71)	1.85 (1.68-2.01)

#### Mean Psychological Reactance Scores

All efficacy messages scored below the midpoint for psychological reactance (Table 8). The message with the highest reactance score addressed quitting smoking by switching to vapes (ID C3).

#### Effect of Efficacy Message Framing on Outcomes

##### Overview

Table 9 presents effects of efficacy message framing on PE and reactance. Preliminary analyses indicated that LGBTQ+ status did not produce significant main or interaction effects across any outcome (results not shown).

**Table 9.** Effect of efficacy message framing on perceived effectiveness (PE) and reactance: findings from an online survey of US young adults reporting ever using both cigarettes and nicotine vapes conducted between February and April 2022 (N=286)^a^.

	Mean (95% CI)	*P* value (vs reference)^b^	*P* value (vs switch)^b^
Main effects of message framing on PE-quit			
Quit all smoking and vaping (reference)	3.11 (2.93-3.29)	—^c^	—
Switch to vaping or NRT^d^	2.23 (2.08-2.39)	*<.001*	—
Quit smoking and switch to vaping or NRT	2.08 (1.94-2.23)	*<.001*	.33
Main effects of message framing on PE-switch			
Quit all smoking and vaping (reference)	2.14 (1.98-2.29)	—	—
Switch to vaping or NRT	3.07 (2.92-3.23)	*<.001*	—
Quit smoking and switch to vaping or NRT	3.22 (3.06-3.38)	*<.001*	.38
Interaction effects of message framing on reactance, by current use status^e^: reactance—current nonuse			
Quit all smoking and vaping (reference)	1.77 (1.47-2.08)	—	—
Switch to vaping or NRT	2.43 (2.07-2.78)	*.006*	—
Quit smoking and switch to vaping or NRT	2.22 (1.82-2.62)	.15	.77

a A total of 286 young adults viewed 3 randomly selected textual health messages (of N=12 messages) with differing message framing. Linear regression models with generalized estimating equations were used to model differences in outcome variable means by message framing (“Quit all smoking/vaping”, “Switch to vaping or nicotine replacement therapy (NRT)”, and “Quit smoking and switch to vaping or NRT”). All models controlled for baseline differences. When no interactions were identified, statistically significant main effects models without interaction terms are reported.

b *P* values were calculated using Wald tests. We assessed statistical significance for overall main effects (6 outcomes×2 moderators) using a Bonferroni-corrected α of 0.004 to control for familywise error rate. When there were significant main effects, post-hoc pairwise comparisons were conducted using Sidak adjustment, with significant differences indicated in italics format (*P*<.05).

c Not applicable.

d NRT: nicotine replacement therapy.

e Significant interactions between message type and nicotine/tobacco use status were identified and decomposed via simple main effects within each level of the moderator. To minimize family-wise error at the decomposition stage, we evaluated simple main effects against a Bonferroni-corrected α of 0.0125 (adjusting for the 4 moderator levels); only strata reaching this significance threshold are displayed. Post-hoc pairwise comparisons were conducted using Sidak adjustment, with significant differences indicated in italics format. All participants reported ever using both cigarettes and nicotine vapes; participants were categorized by self-reported current use of cigarettes and nicotine vapes in the past 30 days as follows: current non-use of cigarettes or nicotine vapes, current nicotine vape vaping only, current cigarette smoking only, or current dual use of cigarettes and nicotine vapes.

##### PE-Quit

We found no significant interaction effects. Main effects models revealed that message framing was associated with PE-quit (*P*<.001 at the Bonferroni-corrected α=0.004). Sidak-adjusted pairwise comparisons showed that participants rated candidate “switch” messages (mean 2.23, 95% CI 2.08-2.39) and candidate “quit and switch” messages (mean 2.08, 95 CI 1.94-2.23) less effective for motivating people to quit all smoking and vaping than “quit” messages (mean 3.11, 95% CI 2.93-3.29; *P*’s<.001).

##### PE-Switch

We found no significant interaction effects. Main effects models revealed that message framing was associated with PE-switch (*P*<.001 at the Bonferroni-corrected α=0.004). Sidak-adjusted pairwise comparisons showed that participants rated candidate “switch” (mean 3.07, 95% CI 2.92-3.23) and candidate “quit and switch” (mean 3.22, 95% CI 3.06-3.38) messages as more effective for motivating people to switch to vaping than “quit” messages (mean 2.14, 95% CI 1.98-2.29; *P*’s<.001).

##### Reactance

We observed a significant interaction between message framing and current nicotine or tobacco use status (*P*=.04). Simple effects decomposition revealed that framing significantly influenced PME-smoking scores among young adults who reported no current smoking or vaping (*P*=.006). Within this group, Sidak-adjusted pairwise comparisons showed that those viewing candidate “switch” messages (mean 2.43, 95% CI 2.07-2.78) reported higher reactance scores than those viewing the “quit” messages (mean 1.77, 95% CI 1.47-2.08; *P*=.006).

## Discussion

### Principal Findings

This mixed methods formative study examined young adults’ responses to standard and comparative candidate messages about cigarette smoking and nicotine vaping, including variation by LGBTQ+ identity and product use status. Overall, both standard and comparative candidate messages were perceived as more effective than regulatory referent messages for influencing smoking-related perceptions, with standard messages generally performing best across outcomes. Psychological reactance was low across message types, suggesting limited counterarguing in response to these text-only stimuli [42,43]. Messages emphasizing exposure to toxic constituents and physical health harms (eg, heart disease and cancer) were consistently rated as most effective.

Importantly, evidence supporting the relative effectiveness of comparative risk messaging was limited and outcome-specific. While comparative messages were associated with higher PE for influencing smoking among young adults reporting current dual use, comparative messages did not demonstrate greater effectiveness than standard messages overall and were less effective for influencing vaping-related perceptions. Our findings suggest that comparative messaging may influence smoking-related perceptions among specific subgroups but do not provide clear evidence of consistent advantages over standard risk messaging.

Qualitative findings provide important context for interpreting these patterns. Participants expressed mixed reactions to comparative messaging. Some viewed these messages as useful for harm minimization, particularly in encouraging reduced cigarette use or substitution of cigarettes with vapes. However, others raised concerns that comparative framing may unintentionally normalize vaping, sustain nicotine use, or be misinterpreted as endorsing vaping as “safe” rather than “lower harm than cigarettes.” These concerns highlight the importance of developing health messages that clearly distinguish between “lower harm” and “no harm” and underscore the need for careful testing and implementation of comparative risk messages [44], particularly in communications targeting heterogeneous young adult populations.

Participants also highlighted the importance of message novelty and content specificity. While messages describing well-established harms (ie, “known truths”) performed well in quantitative analyses, qualitative data suggested that repeated exposure to familiar health risks may reduce engagement among young adults. Messages presenting less familiar information (eg, hypertension and toxic constituents) were perceived as more engaging and credible. Information about nonrespiratory health risks shared by smoking and vaping was particularly “shocking” to participants, especially among LGBTQ+ young adults. This finding was surprising; while fear appeals have commonly been used in tobacco public education campaigns and found to be effective [45,46], contemporary studies suggest that youths and young adults are skeptical of these tactics [47]. Messages on nicotine addiction were perceived as least effective, a finding that aligns with contemporary studies [48-52]. However, participants were more receptive to addiction messaging when paired with novel or contextualized information (eg, risks associated with occasional use).

Efficacy messaging also emerged as a critical influence in shaping message receptivity. Qualitatively, participants preferred messages that acknowledged their ambivalence about quitting all nicotine and provided actionable guidance in a nondirective tone. Encouraging, autonomy-supportive language was viewed as more acceptable than directive or prescriptive messaging, consistent with prior research [47]. At the same time, messages promoting switching to vaping were perceived by some participants as “permissive” and potentially reinforcing nicotine use. These responses highlight a central tension in harm reduction messaging—balancing accurate communication of comparative risks while avoiding unintended consequences (ie, uptake among nicotine-naïve young adults or ongoing use among young adults engaged in mono- or dual-product use).

Our study also highlights the importance of considering developmental differences within the young adult population. Both qualitative findings and quantitative analyses indicated meaningful differences across age groups, with emerging young adults (age 18‐25 years) demonstrating distinct preferences for message tone (encouraging) and content (novel and short-term harms and “off-ramp” messaging). Additionally, younger participants exhibited higher levels of psychological reactance, suggesting that developmental stage may shape how young adults interpret and respond to tobacco risk messages. Prior evidence suggests that emerging young adults may respond to health messages and warnings differently than older, more established young adults [53]; accordingly, future research should explicitly examine how comparative message effects vary across distinct developmental groups.

A key strength of this formative research was the systematic development and testing of candidate messages across qualitative and quantitative phases, allowing triangulation of findings. Our focus on LGBTQ+ young adults supports equity-oriented tobacco research by identifying messaging strategies that may be uniquely salient for this group. Although quantitative analyses did not find evidence of differences by LGBTQ+ status, qualitative data revealed unique thematic patterns. This divergence both underscores the value of qualitative methods for capturing identity-specific nuances and highlights considerations for further investigation. For example, it is possible that standard and comparative messaging approaches resonate broadly across young adults (regardless of LGBTQ+ identity), which could be experimentally tested with larger samples that support subgroup analyses.

### Limitations

Limitations should be considered when interpreting findings. First, convenience sampling through online platforms may have resulted in a young adult sample that is more engaged with or receptive to health research, potentially elevating PME and attenuating reactance relative to broader or higher-risk populations. However, consistency of themes across qualitative and quantitative phases suggests that underlying mechanisms (ie, how young adults process messages) are robust. Second, although mixed-effects models accounted for the incomplete-block design, message-level estimates are constrained by the number of observations and formative nature of the study. Third, our sample included young adults who reported ever smoking and vaping; future studies should use representative samples and focus on at-risk user groups (eg, nicotine naïve users) to better assess population-level effects, including unintended consequences of exposure to comparative messages. Given documented increases in dual use among young people [54], tobacco communication studies should also consider the unique developmental needs and message preferences of emerging (age 18‐25 years) versus established young adults (age 26‐35 years). Fourth, text-only stimuli may underestimate real-world reactance, as visual elements commonly used in health communications may elicit stronger emotional and defensive responses among viewers [55-57]. However, results from a recent meta-analysis also suggest that visual elements may reduce reactance by lowering the cognitive load required for information processing [58]. Finally, this study measured PME and not behavioral outcomes. Although evidence suggests that PME is useful for formative message testing [59,60] and PME predicts both tobacco quit intentions and cessation behavior [61], there is debate regarding PME as a proxy for actual message effectiveness (ie, behaviors) [62,63]. Accordingly, findings should be interpreted as formative and hypothesis-generating.

### Conclusions

This mixed methods formative study contributes to understanding how young adults perceive risk and efficacy messages about cigarette smoking and nicotine vaping. Findings suggest that standard risk messaging performs consistently well across outcomes, while evidence supporting comparative risk messaging is more limited and context-specific. Comparative messages may influence smoking-related perceptions among young adults engaged in dual use, but may also introduce unintended consequences, including normalization of vaping or sustained nicotine use. Regardless of framing, participants responded most positively to messages that conveyed credible and specific health harms, especially novel or less familiar information (eg, heart disease and consequences of occasional use). At the same time, qualitative findings highlight the importance of pairing risk information with clear and actionable efficacy messaging that acknowledges ambivalence about quitting and specific behavioral recommendations. Taken together, findings suggest that the health communications targeting young adults at risk for dual use should prioritize clarity, accuracy, and careful framing of harm information. Campaigns should clearly distinguish “lower harm” from “no harm,” while supporting young adults’ autonomy through informed decision-making. As noncombusted nicotine products continue to enter the tobacco marketplace, future experimental research is needed to assess the behavioral impact of comparative risk messaging, particularly among nicotine-naïve young adult populations, and to evaluate how message effects vary across developmental stages and user groups.

## Supplementary material

10.2196/89822 Multimedia Appendix 1 Messages shown to focus group participants and messages developed after focus groups and tested in interviews and online survey.

## Abbreviations

FDA: Food and Drug Administration
LGBTQ+: lesbian, gay, bisexual, transgender, and queer
LR: likelihood ratio
PE: perceived effectiveness
PME: perceived message effectiveness
