# Supplementary material for: Developing Tobacco Risk Communications for Young Adults Susceptible to Dual Use of Combustible Cigarettes and Nicotine Vapes: Mixed Methods Study
Source: JMIR Form Res. 2026 Aug 18;10:e89822. doi: 10.2196/89822 (PMC13485299; doi:10.2196/89822)
Supplement: Supplementary file 1 [file formative-v10-e89822-s001.pdf]

Table S1. Messages shown to Focus group participants

#	Type	Message
T1a	Candidate Standard	Cigarette smoke contains 69 cancer-causing chemicals.
T1b	Candidate Standard	Cigarette smoke contains 69 chemicals known to cause cancer.
T2a	Candidate Standard	Cigarette smoke AND e-cigarette vapor contains cancer-causing chemicals, including formaldehyde.
T2b	Candidate Standard	Cigarette smoke AND e-cigarette vapor contains chemicals that cause cancer, including formaldehyde.
T3	Candidate Standard	E-cigarette vapor and tobacco smoke contains carbon monoxide–a deadly toxin that deprives your heart of oxygen.
P1	Candidate Standard	Smoking cigarettes increases your risk for lung, head and neck, and other tobacco related cancers.
P2	Candidate Standard	1 in 5 people die each year from smoking cigarettes.
P3a	Candidate Standard	Using both e-cigarettes and traditional cigarettes can increase your risk for cardiovascular disease.
P3b	Candidate Standard	Using both e-cigarettes and traditional cigarettes can increase your risk for heart disease.
P4a	Candidate Standard	Dual users of traditional cigarettes and e-cigarettes experience recurring respiratory issues—including wheezing, coughing, and excess phlegm.
P4b	Candidate Standard	Use e-cigarettes and traditional cigarettes can increase your risk for multiple respiratory issues—including wheezing, coughing, and excess phlegm.
P4c	Candidate Standard	Young people who vape nicotine and smoke cigarettes experience recurring respiratory issues—including wheezing, coughing, and excess phlegm.
P4d	Candidate Standard	Young adults who vape and smoke cigarettes experience recurring respiratory issues—including wheezing, coughing, and excess phlegm.
P4e	Candidate Standard	Smokers who also vape experience recurring respiratory issues—including wheezing, coughing, and excess phlegm.
A1a	Candidate Standard	Approximately 2 in 3 cigarette smokers are dependent on nicotine.
A1b	Candidate Standard	Approximately 2 in 3 smokers are addicted to nicotine.
A1c	Candidate Standard	Approximately 2 in 3 tobacco users are nicotine dependent.
A2a	Candidate Standard	All tobacco products contain nicotine—a highly addictive chemical.
A2b	Candidate Standard	All tobacco products contain nicotine—an addictive chemical.
A2c	Candidate Standard	All tobacco products contain nicotine—an addictive chemical that causes insomnia.
Ad	Candidate Standard	All tobacco products contain nicotine—an addictive chemical that increases heart rate and blood pressure.
RRT1a	Candidate Comparative	Cigarettes contain 9 times more cancer-causing chemicals than e-cigarettes.
RRT1b	Candidate Comparative	Cigarettes contain 9 times more cancer-causing chemicals than nicotine vapes.
RRT1c	Candidate Comparative	There are 9 times more cancer-causing chemicals in cigarettes than nicotine vapes.

RRT2	Candidate Comparative	Cigarettes contain more cancer-causing chemicals— like formaldehyde — than e-cigarettes.
RRT3a	Candidate Comparative	Carbon monoxide-—a deadly toxin that deprives your heart of oxygen-—is higher in dual users of traditional cigarettes and e-cigarettes.
RRT3b	Candidate Comparative	Carbon monoxide-—a deadly toxin that deprives your heart of oxygen-—is higher in people who smoke and vape nicotine.
RRT3c	Candidate Comparative	Carbon monoxide-—a deadly toxin that deprives your heart of oxygen-—is higher in people who smoke cigarettes and vape.
RRP1	Candidate Comparative	Smoking cigarettes and other tobacco products can increase your risk for lung, head and neck, and other tobacco related cancers.
RRP2	Candidate Comparative	1 in 5 people die each year from smoking cigarettes and other tobacco products.
RRP3	Candidate Comparative	Smokers who also vape nicotine are more likely to develop cardiovascular disease.
RRP4a	Candidate Comparative	Dual users of traditional cigarettes and e-cigarettes experience more respiratory issues—including wheezing, coughing, and phlegm—than people who use e-cigarettes only.
RRP4b	Candidate Comparative	People who smoke and vape experience more respiratory issues—including wheezing, coughing, and phlegm—than people who use e-cigarettes only.
RRA1	Candidate Comparative	People who vape and use cigarettes are 7 times as likely to be addicted to nicotine than those who only vape.
RRA2	Candidate Comparative	Both combustible tobacco and e-cigarettes contain nicotine—a highly addictive chemical.
Q1a	Efficacy	Quit smoking and vaping to reduce your risk of cancer.
Q1b	Efficacy	Quit all nicotine and tobacco products to reduce your risk of cancer.
Q2a	Efficacy	Quit smoking and vaping to reduce your risk of cancer.
Q2b	Efficacy	Quit smoking and vaping now to reduce your risk of cancer.
Q2c	Efficacy	Quit smoking and vaping today to reduce your risk of cancer.
Q3a	Efficacy	Quit smoking and vaping to reduce your cancer risk.
Q3b	Efficacy	Quit smoking and vaping to reduce your risk of cancer.
Q3c	Efficacy	Quit smoking and vaping to reduce cancer risk.
Q4	Efficacy	Quit smoking and vaping to reduce your risk of toxic exposure.
Q5	Efficacy	Quit smoking and vaping to keep your lungs healthy.
Q6	Efficacy	Quit smoking and vaping to protect your lungs.
Q7	Efficacy	Quit smoking and vaping to keep your heart healthy.
Q8	Efficacy	Quit smoking and vaping to protect your heart.
S1	Efficacy	Switch to e-cigarettes only to……
S2	Efficacy	Switch to vaping only to….

S3	Efficacy	Switch to nicotine vaping only to….
C1a	Efficacy	If you’re not ready to quit, switch to e-cigarettes only.
C1b	Efficacy	Not ready to quit? Switch to e-cigarettes only
C2a	Efficacy	If you’re not ready to quit, switch to e-cigarettes only.
C2b	Efficacy	If you’re not ready to quit, switch to vaping only.
C2c	Efficacy	If you’re not ready to quit, switch to nicotine vapes only.
C3a	Efficacy	If you’re not ready to quit, use e-cigarettes only.
C3b	Efficacy	If you’re not ready to quit, use nicotine vapes only.
C3c	Efficacy	Not ready to quit? Use….

****NOTE: For Efficacy messages, “Quit” statements [Q] emphasize quitting all smoking and vaping, “switch” statements [S] emphasize using another source of nicotine other than smoking, combo “quit plus switch” statements [C] emphasize quitting only combusted products.*

Table S2. Messages developed after Focus groups and tested in Interviews and Online Survey

#	Type	Message
7	Regulatory Standard	Cigarettes and vapes contain nicotine. Nicotine is an addictive chemical.
6	Candidate Standard	Even occasional smoking and vaping can lead to nicotine addiction.
10	Candidate Standard	Can't sleep? Nicotine in cigarettes and vapes can lead to sleep problems, including insomnia.
4	Candidate Standard	Nicotine doesn't cause cancer, but toxic chemicals in e-liquid and tobacco can.
2	Candidate Standard	Most people think smoking cigarettes and vaping nicotine are harmful to your health. They're right.
12	Candidate Standard	Can't find your keys? Nicotine in cigarettes and vapes can harm your brain and lead to memory loss.
8	Candidate Standard	Smoking cigarettes and vaping nicotine damages your DNA and increases your risk for cancer.
5	Candidate Standard	Using cigarettes and nicotine vapes exposes you to heavy metals— like arsenic and lead.
11	Candidate Standard	Wheezing lately? Smoking cigarettes and vaping nicotine increases your risk for lung problems.
3	Candidate Standard	Cigarettes and some nicotine vapes contain formaldehyde, a chemical that causes cancer.
9	Candidate Standard	Smoking cigarettes and vaping nicotine increases your risk for heart disease.
1	Regulatory Comparative	Nicotine vapes are not a safe alternative to cigarettes.
24	Candidate Comparative	Can't find your keys? Research suggests that vaping nicotine can improve attention and memory during smoking abstinence.
22	Candidate Comparative	Can't sleep? People who vape and smoke report more sleep problems than those who only vape.
14	Candidate Comparative	Many people think vaping nicotine is just as harmful as smoking cigarettes. Truth is, nicotine vapes are less harmful than cigarettes.
19	Candidate Comparative	Nicotine vapes are addictive, but people who vape and smoke tobacco are 7 times more likely to be addicted to nicotine than those who only vape.
18	Candidate Comparative	Vaping heats nicotine, resulting in lower levels of harmful chemicals than burned tobacco in cigarettes.
13	Candidate Comparative	Vaping nicotine is harmful to your health, but it is less harmful than smoking.
21	Candidate Comparative	People who smoke cigarettes and vape nicotine have a higher risk of developing heart disease than those who only vape.
17	Candidate Comparative	Using cigarettes and nicotine vapes exposes you to more heavy metals—like arsenic and lead—than if you only vape.
23	Candidate Comparative	Wheezing much? People who smoke cigarettes and vape nicotine report more lung problems than those who only vape.
16	Candidate Comparative	Many people think nicotine in vapes and cigarettes causes cancer. Truth is, toxic chemicals in burning cigarettes are more likely to cause cancer.
15	Candidate Comparative	Cigarettes contain more cancer-causing chemicals—like formaldehyde—than nicotine vapes.

20	Candidate Comparative	Smoking cigarettes can increase your risk for lung, head, and neck cancer. Using nicotine vapes only may decrease your cancer risk.
Q1	Efficacy	Quit all smoking and vaping to reduce serious risks to your health.
Q2	Efficacy	Quit all smoking and vaping to reduce your cancer risk.
Q3	Efficacy	Quit smoking and vaping completely to reduce your exposure to toxic chemicals.
Q4	Efficacy	Ready to quit smoking and vaping completely? Manage your cravings with nicotine replacement therapy.
S1	Efficacy	Switching completely from smoking to vaping nicotine can reduce serious risks to your health.
S2	Efficacy	Switching completely from smoking to vaping nicotine can reduce your cancer risk.
S3	Efficacy	Switching from smoking to vaping only to reduce your exposure to toxic chemicals.
S4	Efficacy	Manage your smoking cravings with nicotine replacement therapy.
C1	Efficacy	Not ready quit completely? Quit smoking all tobacco, and use nicotine vapes only to reduce serious risks to your health.
C2	Efficacy	If you're not ready to quit all nicotine, switching completely to vaping may reduce your cancer risk.
C3	Efficacy	Start quitting smoking by switching to nicotine vapes only.
C4	Efficacy	If you're not ready to quit all nicotine, using nicotine replacement therapy can help you manage cravings and quit smoking.

****NOTE: All EFFICACY statements will be paired with an efficacy statement to connect audiences to quit resources as follows: "For free quit resources, visit smokefree.gov or text QUIT to 47848. "Quit" statements [Q] emphasize quitting all smoking and vaping, "switch" statements [S] emphasize using another source of nicotine other than smoking, combo "quit plus switch" statements [C] emphasize quitting only combusted products.*
